# Navigating the AI landscape in SMEs: Overcoming internal challenges and external obstacles for effective integration

**DOI:** 10.1371/journal.pone.0323249

**Published:** 2025-05-27

**Authors:** Roziah Mohd Rasdi, Nordahlia Umar Baki

**Affiliations:** Department of Professional Development and Continuing Education, Faculty of Educational Studies Universiti Putra Malaysia, Serdang, Selangor, Malaysia; Islamic Azad University Urmia Branch, IRAN, ISLAMIC REPUBLIC OF

## Abstract

Artificial intelligence (AI) integration in small and medium-sized enterprises (SMEs) is hampered by a variety of internal and external challenges. This article analyses these hurdles and underlines the need of an adaptation framework designed particularly for SMEs. The study collects primary data by conducting detailed interviews with representatives from six different SMEs in distinct sectors. The results indicate substantial internal obstacles, including reluctance to change, fear of job displacement by technology, and restricted resources, all of which impede the incorporation of AI. SMEs encounter a dynamic technological landscape, stringent regulatory requirements, and intense rivalry externally, necessitating agile and prompt strategy adjustments, a challenge often faced by SMEs. Ultimately, this research highlights the need of creating AI implementation plans that are customized to the distinct requirements and situations of SMEs. More adaptable and supportive legislative frameworks are essential to assist these enterprises in efficiently using AI and staying competitive in the digital era. This study contributes to the current discourse on technological progress in SMEs and establishes the foundation for next policies and initiatives designed to enhance their competitive edge using AI technology.

## Introduction

The integration of artificial intelligence (AI) in contemporary business environments has significantly impacted small and medium enterprises (SMEs). AI, which mimics human intelligence processes such as learning, reasoning, and problem-solving, is widely applied to enhance operational efficiency, drive innovation, and provide data-driven insights, thereby enabling businesses to optimize various operations [1]. In Malaysia, SMEs are defined by specific thresholds, with sales turnover not exceeding RM50 million or full-time employees not exceeding 200, varying between manufacturing and service sectors [2].

Research suggests that AI can substantially increase productivity, enhance decision-making, and empower SMEs to compete globally. According to [3] and [4], AI helps businesses boost operational efficiency, innovate new products, and enhance market dominance. In developing countries like Malaysia, AI is critical for economic growth and gaining a competitive edge in the digital age. The Malaysian government’s support for Industry 4.0 initiatives reflects its commitment to creating a technologically advanced economy where SMEs play a vital role [5]. AI is pivotal in boosting productivity and fostering innovation, aligning with global trends that link digital skills to business success.

However, integrating AI into SMEs involves more than just adopting new technologies. It requires a comprehensive transformation of existing corporate processes, cultures, and infrastructures. This complex transformation process presents several internal and external challenges that may impede progress and long-term success. Financial constraints and the need for skilled workers are the two primary barriers to AI integration in SMEs [6,7]. Additionally, many SMEs struggle with outdated technological infrastructures incompatible with advanced AI applications, posing significant threats to their sustainability and limiting the adoption pace [8].

Moreover, effective sustainability efforts are hampered by high costs, limited resources, and knowledge gaps, which deter the adoption of sustainable practices [9]. Strategic sustainability initiatives and overall quality management are often lacking, further complicating AI integration. SMEs also face challenges from rapidly evolving AI technology, necessitating continuous adaptation and learning. [10] notes that navigating complex data protection regulations and AI ethics can be particularly burdensome and costly for smaller firms. Additionally, the digital divide between urban and rural areas exacerbates access disparities, preventing equal benefits from AI technology across all SMEs [11]. This gap leads to limited computer skills, low technology adoption, and restricted business growth due to poor connectivity [12]. Resistance to adopting new technology within SME cultures, driven by concerns about displacement and disruption, further complicates AI integration [13].

Despite the recognized benefits of AI, there is a notable lack of specialized, evidence-based strategies tailored for SMEs, particularly those in developing regions [14]. Research often focuses on AI’s broader impacts and strategies for larger companies, neglecting the unique circumstances, capabilities, and needs of SMEs [15]. Studies on AI in large firms typically overlook the distinct issues, capacities, and demands of SMEs, resulting in a significant gap in customized approaches that could effectively assist SMEs in navigating their unique AI adoption environments [16]. Additionally, there is limited research on the long-term impacts of AI on SMEs, highlighting the need for more in-depth studies to monitor the integration process over time and identify potential problems.

This study aims to explore the challenges and obstacles SMEs face in adopting AI and offers practical solutions tailored to their specific circumstances. The objective is to raise awareness among policymakers, researchers, and business leaders about the importance of AI integration in SMEs, particularly in managing technology and fostering SME growth. Studies such as those by [17] and [18] have shown that proactive AI deployment by SMEs can contribute to achieving the Sustainable Development Goals (SDGs). The SDGs emphasize creating a sustainable future for all, with SMEs playing a critical role in achieving these goals. By leveraging AI, SMEs can take a leading role in advancing sustainable development, ensuring that the benefits of technology are distributed fairly and inclusively.

While AI integration in SMEs offers numerous advantages, it is crucial to acknowledge its connection to broader global goals, such as the SDGs. [17] stress that SMEs can make significant contributions to these goals by actively incorporating AI into their operations. The SDGs prioritize a collaborative approach to achieving a sustainable future, highlighting the crucial role SMEs play in this endeavor. Through AI, SMEs not only enhance their competitiveness but also promote sustainable growth, ensuring fair and inclusive access to technological advancements. Therefore, AI integration extends beyond individual business success, positioning SMEs within the broader context of sustainable development and ethical innovation. This underscores the importance of aligning AI integration strategies with both business growth and societal well-being.

### AI empowerment for SMEs

Integrating AI into SMEs represents a transformative shift in modern business, offering opportunities to enhance operational efficiency, innovate product offerings, and expand market reach. AI significantly boosts productivity, improves decision-making, and enhances global competitiveness for SMEs by analyzing vast amounts of data and tailoring strategies to dynamic market needs [4,19,20]. During the COVID-19 pandemic, AI proved vital for SMEs, enabling automation, optimizing logistics, and supporting remote work, thereby maintaining business continuity and employee productivity [21].

However, the road to effective AI integration is fraught with challenges. Financial constraints and poor creditworthiness hinder SMEs from securing the necessary funds for AI investments [22,23]. The high costs of AI infrastructure and the lack of internal expertise further complicate this process [8,24]. Internal resistance, concerns about job loss, and navigating external AI regulations and ethics pose additional barriers [10,13].

Despite these challenges, the benefits of AI for SMEs are immense. Strategic partnerships with tech companies and academic institutions can provide access to cutting-edge innovations and expertise. Government support, including financial incentives, can also play a crucial role in facilitating AI adoption [5]. Ultimately, by strategically integrating AI, SMEs can enhance their competitiveness, ensure long-term sustainability, and secure a strong position in the global marketplace. Empowered by AI, SMEs can overcome operational inefficiencies, continuously innovate, and adapt more effectively to environmental and market changes, achieving a significant competitive advantage.

### Theorizing the integration of AI in SMEs amidst challenges

Integrating Resource-Based View (RBV) with PESTEL analysis provides SMEs with a structured method for AI adoption, enabling them to align internal strengths with external challenges. By leveraging strategic planning and AI capabilities, SMEs can manage external threats and capitalize on opportunities, enhancing resilience and competitiveness in an AI-driven market [25].

#### Resource-based view.

The Resource-Based View (RBV) framework highlights the importance of combining strategic resources and capabilities to generate long-term competitive advantages, emphasizing internal capabilities in promoting AI adoption and enhancing SMEs’ performance [26]. By leveraging internal resources such as entrepreneurial talents, human resources, technical competence, innovative capabilities, and social networks, SMEs can effectively embrace AI technologies to boost their competitiveness in the digital age [27].

[28] suggest that SMEs can cultivate success and resilience by effectively navigating and maximizing these internal resources to support innovation and development. This aligns with [15] findings, which emphasize that long-term competitive advantage is based on distinctive, valuable, and difficult-to-replicate resources and capabilities. For SMEs, often constrained by limited financial and human resources, the RBV framework provides a path to success by focusing on unique capabilities.

Research on small manufacturing firms in Portugal underscores the significance of internal information systems, technical capabilities, and management attitudes towards technology adoption [15]. SMEs that develop their expertise, knowledge, and strategic management perspectives are better positioned to succeed in AI integration, consistent with the RBV principle that emphasizes distinctive skills.

The RBV framework assists SMEs in identifying and developing key capabilities for AI integration. By focusing on areas such as AI competence, data analytics skills, and technological infrastructure, SMEs can enhance their AI readiness and effectiveness. Additionally, the RBV framework helps SMEs utilize their unique resources to establish a competitive edge and deliver value through AI adoption.

**PESTEL analysis**

We applied the PESTEL framework to analyze the external macroenvironmental factors affecting SME operations and performance, focusing on Political, Economic, Social, Technological, Environmental, and Legal aspects [29]. Political factors, such as changes in trade policy or taxation, impact market access and operating costs. Economic variables, including inflation and local economic growth, influence SMEs’ financial health, market demand, and pricing strategies. Social factors, like evolving consumer preferences and demographics, compel SMEs to adjust marketing strategies and products to meet changing customer needs. Technological advancements, such as affordable cloud computing and digital marketing tools, can enhance SME productivity and market reach. Environmental considerations drive SMEs to adopt sustainable practices, such as waste reduction and energy efficiency, to meet customer expectations and legal requirements. Legal compliance with labor laws, health and safety regulations, and intellectual property rights is crucial for smooth SME operations.

By applying PESTEL to AI integration, SMEs can anticipate and mitigate risks while identifying opportunities for innovation and growth. Understanding technology trends through PESTEL enables SMEs to enhance operations, improve customer experiences, and maintain competitiveness. Additionally, staying informed about regulatory developments helps SMEs ensure compliance and avoid legal issues related to AI integration [25].

In summary, integrating RBV and PESTEL provides SMEs with a comprehensive framework to address internal capabilities and external pressures in AI integration. By leveraging internal resources and strategic planning while considering external factors like regulations, economic trends, and technological advances, SMEs can navigate AI adoption’s complexities, positioning themselves for sustainable growth and competitive advantage in the digital era.

## Methods

This study employed a qualitative research method using a multiple case study design to investigate the experiences of six participants from various SME sectors regarding AI integration. The multiple case study approach allowed for a detailed analysis of real-world events in SME environments, providing in-depth insights into the complexities of AI deployment [30]. This method was chosen to explore SMEs’ experiences with AI, considering both internal and external challenges.

Data were collected through in-depth semi-structured interviews conducted across multiple settings to understand the integration processes and challenges faced by SMEs in adopting AI technology. This approach enabled the capture of detailed, contextual aspects influencing AI adoption by exploring individual experiences within a case study framework [31]. The interviews were designed to be flexible, allowing participants to share their ideas, experiences, and perspectives while following a structured interview protocol. This method is effective in gathering intricate, qualitative insights [32].

The interview guide featured open-ended questions focused on topics such as AI implementation, perceived benefits and challenges, readiness for AI integration, and the impact of government support. Each interview lasted between 60 and 90 minutes, conducted either face-to-face or via online platforms, depending on participant availability. All interviews were audio-recorded and transcribed for analysis with participant consent.

### The research setting

In the context of Malaysian SMEs, the government’s support for AI integration is evident through initiatives like the National Policy on Industry 4.0 (Industry4WRD) and the Malaysia Digital Economy Corporation (MDEC) projects [33,34]. These efforts aim to enhance SME competitiveness through digital transformation, including AI adoption. However, SMEs face challenges such as limited financial resources, a lack of skilled workers, and inadequate infrastructure. MDEC’s Digital Transformation Acceleration Program addresses these issues by providing technical and financial assistance. Nevertheless, the success of AI integration depends on SMEs aligning their goals with available resources and leveraging government support effectively.

### Data collection procedures

The data collection process strategically identified SMEs through websites and colleague referrals, ensuring participants were actively integrating AI. Upon consent, interviews were conducted either at participants’ offices or via online platforms like Zoom, accommodating preferences and logistical constraints. All interviews were recorded and meticulously transcribed for detailed analysis, ensuring the findings were rooted in authentic insights. This approach provided a comprehensive understanding of SMEs’ AI integration experiences and challenges. Flexibility in research design was crucial, fostering an environment conducive to open and informative exchanges, contributing valuable insights to the discourse on AI in SMEs.

### Data analysis

Data were analyzed using Atlas.ti for thematic and comparative analysis, identifying themes, patterns, and recurring concepts. To enhance qualitative rigor, methodological triangulation was employed by integrating data from document analysis (e.g., SMEs’ reports, government policies, etc.), interviews, and observations, ensuring that findings were not solely reliant on interview data. Additionally, inter-coder reliability was maintained through independent coding by multiple researchers, followed by consensus discussions to resolve any differences in coding. Reflexivity, audit trails, and peer debriefing were also applied to check for alternative interpretations and ensuring reliability and trustworthiness [35]. This comprehensive approach ensured the credibility and dependability of the study’s findings.

### Participants

The study strategically involved participants from six SMEs across diverse sectors, including IT, laboratory services, manufacturing, agriculture, healthcare, and robotics, to capture a wide range of perspectives on AI integration (See Table 1). Participants held varied roles such as research officers, executives, engineers, and owners, ensuring a comprehensive understanding of AI adoption across different organizational levels. They were selected based on three criteria: (i) their SMEs had integrated AI, (ii) they were responsible for AI strategy and execution, and (iii) they had at least three years of experience with the firm. This ensured they provided valuable insights into AI implementation challenges. To maintain anonymity, pseudonyms were used, and ethical guidelines were strictly followed, with informed consent obtained from all participants.

**Table 1:** Background of the participants.

Pseudonyms	Education	Position	Sector	Number of Employees
P1	Bachelor of Engineering Technology - BETA, Mechatronics in Automotive	Head Engineer	Information Technology & Services	18
P2	Master of Engineering Technology (Electrical & Electronics)	Research Officer	System Engineering & Energy Lab	19
P3	Bachelor of Engineering - Food Technology and Processing	Quality Control Executive	Food & Beverage Manufacturing	27
P4	Bachelor of Science, Biotechnology	Operation Manager	Agriculture	N/A
P5	Doctor of Medicine (Kursk State Medical University)	Chief Executive Officer (CEO)	Medical Service	10
P6	Bachelor Automation Engineering	Manager/ Automation Engineer	Robotic	17

Beyond the limited sample size, it is crucial to recognize that the inherent nature of qualitative research designs, often prioritizing in-depth understanding over broad generalization, also shapes the scope of these findings. While the strategic sectoral and role diversity offers valuable rich data, the focus on detailed insights from a small number of cases means that statistical generalizability to the wider SME population is inherently constrained by the chosen qualitative approach.

## Findings and discussion

We organised the challenges into internal and external categories to better address areas needing attention for successful AI integration. Internal challenges require organizational adjustments, while external challenges may need advocacy and partnerships. This classification enables targeted interventions. Six themes and 14 sub-themes were identified, as detailed in Table 2, with further explanation provided in the next section.

**Table 2:** SMEs challenges in adopting AI at the workplace.

Issue	Theme	Sub-themes/ descriptions	P1	P2	P3	P4	P5	P6
Internal challenges	Change management and acceptance	Lack of understanding how AI operate	√√		√	√	√	
		The complexity of AI technology					√	√
		Leader mindset		√			√√	
		Fear of job displacement		√				√
	Limited in resource and infrastructure	Lack of compatible facilities	√	√			√	
		Cost of AI deployment			√√	√	√	√
External challenges	Workforce skills gap	Shortage of AI experts	√√	√	√			√
		Skill mismatched	√				√	√
	Market and Competitive Pressures	Talent competition	√	√			√	√
		Advanced technological capabilities		√			√	√
	Regulatory and legal consideration	Regulatory compliance		√			√	√
		Lack of established policy			√		√√	√
	Resource Acquisition Challenges	Lack of government infrastructure			√		√	√
		Insufficient fundings from banks or government bodies	√	√	√	√	√	

* Note: √√ indicates that the participant emphasized this point multiple times during the interview.

### Internal challenges

In SMEs, internal challenges arise from within the organization, impacting its ability to achieve goals. The study identified two key internal challenges: change management and acceptance, and limitations in resources and infrastructure, necessitating strategic interventions.

#### Change management and acceptance.

SMEs face resistance from employees and clients due to a lack of understanding of AI, its complexity, fear of technology and job displacement, and leadership mindset. [31] noted that AI adoption’s complexity sets it apart from other digital technologies, emphasizing the need for customization aligned with an organization’s AI readiness and goals. Participants reported that change management and acceptance issues hinder AI implementation, with [36] highlighting insufficient awareness and understanding as a major challenge, leading to decreased employee engagement with AI. P3, the only female participant, specifically mentioned these challenges.

*“It is true that most worldwide companies have already adopted IR 4.0, AI, and so on, but in Malaysia, we are still behind, one of the reasons being a lack of awareness.”*

AI implementation poses significant challenges for workers, who must adapt to new operational models and cope with the psychological impact of machines taking over their roles. P6, an AI technology service provider, recounted an incident where blue-collar workers, fearing job replacement by robots, resisted by attempting to destroy a cleaning machine in protest.

*“When we first started in 2014, mobile robots were very rare in the manufacturing industry, so we had people who would actually sabotage the robots. Workers would come and damage the robots. They felt like they were suddenly competing with a robot that does not get tired and can run 24/7, which we understand… Recently, one time, I believe one of our teams was deploying it at hospital X. When one cleaner arrived, she turned the robot cleaner off and fled. We understand and can feel the worry and concern because this is a job that she used to do. Suddenly, a robot is doing it much quicker and more efficiently.”*

This finding is in line with [37] study on radiologists, which highlighted the intricacy of AI and concerns about job loss as obstacles to AI implementation. Therefore, organisations need to implement more sensitive and effective change management practices in the workplace when embracing AI technology. [38] highlighted that the success of AI technology depends on workers’ acceptance. The results imply that SMEs should prioritise aligning AI projects with the technology environment to promote easier integration and acceptability among workers [39].

#### Limited resources and infrastructure.

SMEs often face significant challenges in AI integration due to limited financial and human resources and inadequate technology infrastructure [40,41]. [38] note that financial constraints and outdated infrastructure are major barriers, as AI requires substantial upfront investments, ongoing maintenance, and upgrades. [42] emphasizes the need for advanced technological infrastructure—high-speed internet, advanced computing power, and adequate data storage—to support AI, which many SMEs lack, leading to integration and performance issues. P1, a head manager, highlighted the crucial role that infrastructure plays in either supporting or hindering AI integration within an organization.

*“The challenges are where the infrastructure and environment are not ready. Sometimes, not because workers are reluctant to adapt, but because they are not exposed to the infrastructure... When the organisation is located on the outskirts of town or in a rural place, it is challenging to get access to the high and better coverage of infrastructure.”*

In a competitive environment, high-performance AI tools can significantly enhance business performance. However, implementing AI requires substantial resources, posing challenges for SMEs with limited financial capacity [43]. [44] found that SMEs often face financial constraints, limiting investment in technology, which affects employment, productivity, and wages. Both P5 and P2 highlighted that these financial constraints are a major concern for many Malaysian companies due to the high capital required to sustain and advance AI technologies. P2 specifically noted the difficulties in maintaining and upgrading AI systems.

*“I think one of the challenges that all Malaysian companies have right now is the cost of adopting these types of technologies.”*

### External challenges

Recognizing external issues is crucial for successful AI integration in SMEs, as factors like workforce skill gaps, competitive pressures, and regulatory restrictions significantly impact AI implementation’s feasibility and success, further explored in the following sub-headings.

#### Workforce skills gap.

Many SMEs lack employees with AI expertise, leading to a reliance on costly external consultants or the challenge of attracting limited AI talent. Despite financial constraints, SMEs should invest in high-value assets to attract or retain AI professionals, as they often struggle to compete with larger firms due to budget limitations [45,46]. P1 noted that the shortage of AI professionals makes it difficult for SMEs to attract talent, with costs frequently exceeding their budgets.

*“… my challenge is to hire a colleague who has expertise in data, but they are limited, and his fee is very high. Very expensive. So, we ourselves cannot afford it.”*

P5, the CEO of SMEs, who is also a doctor, pointed out that although local institutions are producing more graduates in AI-related subjects, there is a skill mismatch with the organisation’s requirements to work with current AI technology. The rapid advancement of technology leads to constantly evolving skill requirements for AI integration, making it difficult for talents to keep up to date [45]. Consequently, some highly educated graduates are compelled to accept blue-collar jobs to secure employment.

*“A lot of young talent our university provides is very good, but they don’t have the required skills. It is very sad when you hear this. AI software engineer graduated from top universities but works as a food panda driver. It is not right. Something is wrong.”*

#### Market and competitive pressures.

Integrating AI provides a competitive edge by improving operational efficiencies and product offerings, while those slow to adopt risk falling behind early adopters [47]. The competitive environment drives companies to strategically invest in AI and hire top talent, balancing short-term needs with long-term goals [48]. P6 acknowledged that competition from larger firms makes it difficult for SMEs to attract and retain top talent, hindering their growth and limiting their ability to fully leverage AI.

*“It is a challenge to find experts in AI. I mean, to the extent that you hear of companies and major players poaching or stealing workers from each other. Because of the limited supply, it is quite an emerging market.”*

Another challenge faced by SMEs in terms of market pressures is the adoption of advanced technologies, particularly in the context of AI integration [49]. In comparison, larger organisations are more likely to implement cutting-edge technology due to their greater access to resources and larger opportunities, which gives them an advantage in competing with smaller firms, including SMEs. P2 stated that in the digital era, SMEs strive for either the top talent or the most cutting-edge technology when compared to their competitors.

*“Limitation means that the organisation needs professional power. In terms of business, it will cost more for the organisation to compete with... to get the best talent and technology.”*

#### Regulatory and legal considerations.

The study identifies regulatory compliance, lack of established policy, and insufficient government support as key challenges in integrating AI into SMEs. The evolving regulatory and legal framework for AI increases complexity, making it essential for companies to navigate these requirements to avoid penalties. Data privacy and protection are major concerns, as AI systems require substantial data, necessitating compliance with laws like Malaysia’s Personal Data Protection Act (PDPA). This involves obtaining proper consent, implementing robust security measures, and ensuring transparency in AI data use [50]. [51] emphasize that while adhering to these evolving standards is challenging, it is crucial for sustaining operations. P5 suggests that developing consistent policies can help manage legal norms over time. However, P5 also noted that frequent policy changes due to political concerns hinder organizational progress, as SMEs lack stable guidance for advancement. The need for a consistent and clear regulatory environment is critical for supporting SMEs in effectively integrating AI while maintaining compliance.

*“In Malaysia, all the movements start from top to bottom, not bottom to top. So, government policy is important. The stability of the government is very important. The policy cannot change if the government runs the country or if the healthcare policy in AI is like this. And then, when it comes to government B (run the country), because I experienced it from 2017, 2018 and to 2019. It is a totally different policy. Because the different policies have different adoption angles and different funding programs, the policy needs to be in line with the AI.”*

[52], in their study, advised for appropriate policies, regulations, ethical advice, and a legal framework to prevent the misuse of AI in the organisation. At the same time, the comprehensive policy can regulate AI within ethical standards and in the context of human rights’ protection [25]. Similarly, P6 underlined that embracing AI means that businesses, no matter how small or large, must prioritise ethical issues or face more disadvantages than benefits. He then provided examples of what happens when we ignore ethical issues.

*“For example, I am sure you have heard recently that there are these AI image generators that you would give something to, and then it would generate an image. And it caused controversy recently when there was an art competition, and someone used an AI image generator and won first place in the art competition. So, these things will create some concerns, and we are seeing some of that play out. So yeah, of course there are ethical issues; the good thing is that the artist who won first place came up and said, I did not draw this; I used an AI-generating tool, and his reason was that he wants to raise awareness of this issue.”*

#### Resource acquisition challenges.

The final theme identified in this study is resource acquisition challenges, focusing on insufficient funding from banks or government agencies and the lack of infrastructure to support AI integration. SMEs often struggle with limited financial, human, and technological resources, unlike larger companies [53]. This resource shortage impairs their ability to compete effectively and adapt to changing economic conditions [54]. Without sufficient capital, SMEs cannot invest in modern technology and infrastructure upgrades, placing them at a competitive disadvantage in terms of efficiency and output. P4, representing a SME providing technology to farmers, highlighted the difficulty in securing financing from banks or government organizations due to their small size, often making loans inaccessible. As a result, they rely on government subsidies or grants to support their business, but are frequently met with bureaucratic hurdles or insufficient funding. This forced the company to use whatever resources it had to survive the AI integration process, underscoring the significant challenges SMEs face in acquiring the necessary resources to advance and sustain AI adoption.

*“The biggest challenge is funding. Because we are new, getting help from a bank or government agency is difficult and takes years. Sometimes it takes 16 months or more (before being able to get funds). So, if we want to run the operation, I need to find funding for myself, which is the most difficult thing for me because we need to use it immediately.”*

Another group of participants, P1, P2, P3, P4, and P5, emphasised that, besides budget constraints, inadequate infrastructure, such as old equipment or insufficient logistics networks, can hinder operations and restrict expansion potential. P4 believed that the infrastructure should be provided by local authorities or government agencies because this is something that the government can do to facilitate small firms progress in the digital era.

*“Because IKS (refers to SMEs) have limited resources and we do not have or afford to invest in providing better infrastructure, we are often left behind... Infrastructure is something that the government or local authority should provide in order to support our expenditures.”*

## Conclusion

The adoption of AI in SMEs presents numerous opportunities alongside significant challenges. This study explores the internal and external obstacles that hinder effective AI deployment, emphasizing the need for tailored strategies and frameworks to support SMEs in this technological transformation. Customized approaches that leverage internal resources are essential for recognizing opportunities for growth and innovation while also predicting and mitigating risks and external challenges. Understanding both internal and external factors that impede AI deployment in SMEs is crucial for success.

Internal challenges such as change management, employee acceptance, and limited resources and infrastructure are key barriers to successful AI integration within SMEs. These challenges require strategic interventions to align internal capabilities with AI adoption goals. On the external front, workforce skill gaps, competitive pressures, regulatory and legal considerations, and resource acquisition challenges further complicate the AI adoption process. Overcoming these obstacles is vital for boosting SMEs’ competitiveness, innovation capacity, and overall efficiency, ensuring their long-term viability and success in the digital economy.

AI implementation in SMEs differs from large corporations primarily due to financial and human resource limitations, leading to incremental and customized AI adoption. Large corporations typically have more substantial budgets to support comprehensive AI systems and talent acquisition [9], allowing them to implement extensive systems across multiple departments [54]. SMEs, on the other hand, prioritize flexibility and scalability, adopting AI in a more phased manner.

Internal factors like leader mindset and change management significantly influence AI adoption in SMEs, necessitating tailored approaches to manage these dynamics effectively [55]. SMEs benefit from more direct leadership involvement, enabling quicker decision-making and more adaptive AI strategies compared to the hierarchical structures in large corporations that can slow down AI implementation [31].

Regulatory navigation and competitive pressures pose additional challenges for SMEs. While larger corporations have dedicated compliance teams, SMEs may need external support to ensure regulatory compliance [56]. Moreover, SMEs face intense pressure to innovate to remain competitive, unlike larger companies that enjoy established market influence [57].

Despite these challenges, SMEs have the potential to significantly benefit from AI and IoT technologies if they can effectively navigate the unique adoption barriers and opportunities within their operational contexts [58]. In summary, while large corporations leverage their resources and organizational structure for AI implementation, SMEs rely on their agility and flexibility to develop customized solutions. This requires distinct strategies to ensure effective AI integration, tailored to their specific needs and constraints.

## Implications for theory and practice

This study contributes to the debate on AI integration in SMEs by highlighting the unique challenges small businesses face, distinct from those encountered by larger enterprises. Traditional technology adoption models often focus on large, resource-rich companies with complex infrastructures, while SMEs operate under constraints like limited financial resources, expertise, and structured processes. The study emphasizes the importance of positioning AI strategies within SMEs’ unique operational and cultural dynamics.

To systematically understand these dynamics, the study integrates the Resource-Based View (RBV) and PESTEL analysis. RBV emphasizes leveraging internal resources and competencies, suggesting that SMEs can achieve a competitive advantage by focusing on their specialties and agility. PESTEL helps SMEs navigate external factors like regulatory changes and technological advancements, providing a comprehensive framework for strategically implementing AI. This integration challenges the one-size-fits-all approach prevalent in existing literature, offering SMEs a tailored approach to align internal readiness with external opportunities and threats [25,54].

### For companies/ SMEs

The study suggests that SMEs should adopt a comprehensive approach to AI integration, focusing on enhancing internal resources, fostering a flexible corporate culture, and navigating complex regulatory environments. Internal resource enhancement involves investing in capability development, such as upskilling employees, upgrading technical infrastructure, and promoting innovation [39]. SMEs can assess their AI adoption stage by creating a checklist of key criteria, including digital infrastructure, data analytics capabilities, AI-skilled personnel, and cultural readiness for innovation. This assessment helps identify strengths and weaknesses, guiding targeted investments.

To support AI efforts, SMEs should establish continuous learning programs, upgrade IT infrastructure, and encourage business leaders to champion AI initiatives. Cultivating an adaptive company culture is crucial, as resistance to change often hinders AI adoption. Effective change management strategies, clear communication of AI benefits, and a phased deployment approach can facilitate this cultural shift [38]. This strategy ensures SMEs are better equipped to integrate AI, leading to enhanced competitiveness and operational efficiency.

### For the government

The government has a vital role in promoting the use of AI especially when it comes to adhering to data protection and privacy laws due to the intricate regulations and difficulties that SMEs encounter. SMEs have the potential to gain substantial advantages from legislative incentives that provide rewards to those that fulfil certain criteria related to their preparedness for AI. These incentives may consist of grants, tax incentives, or subsidized training programs that empower SMEs to embrace AI technology without being excessively burdened by the initial financial costs. By using this approach, the government may provide a conducive environment that promotes innovation while guaranteeing that the incorporation of AI is carried out in a manner that adheres to ethical and legal frameworks [10].

## Limitations and recommendations for future study

The present study has limitations that could be addressed in future research. While the sample size of six SMEs from various industries offers valuable insights, it may not fully capture the diverse experiences of all SMEs. Indeed, the small sample size significantly restricts the statistical generalizability of the findings, a limitation further compounded by the qualitative research design employed. Qualitative studies, like this one, are typically designed to achieve depth and nuanced understanding of a phenomenon within specific contexts, rather than to produce broadly generalizable statistical claims. Therefore, the goal is not to create findings that are statistically representative of all SMEs, but rather to offer rich, contextualized insights into the AI integration experiences of these particular six organizations, limiting the extent to which findings can be broadly applied.

A larger, more varied sample would provide a more comprehensive understanding of the challenges and opportunities associated with AI integration in SMEs [30]. Furthermore, even with the commendable sectoral diversity within this qualitative framework, having only a few SMEs representing each broad sector means the study may not fully capture the considerable heterogeneity that exists within each industry, and this inherent characteristic of qualitative research, with its focus on depth over breadth, further influences the transferability of these insights beyond the studied cases.

Additionally, the study’s qualitative approach, though detailed, could be enhanced by incorporating quantitative methods. Quantitative research could statistically validate the findings and offer a broader perspective on AI integration trends across a wider sample of SMEs.

Another limitation is the study’s geographical focus, which centers on SMEs in a specific environment. Future research could explore the impact of regional and cultural factors on AI adoption in SMEs across different regions [31]. Investigating the distinct challenges faced by SMEs in rural versus urban settings would be particularly beneficial, given the disparities in infrastructure, access to skilled labor, and market dynamics.

Moreover, future studies could employ longitudinal methodologies to monitor the development and long-term effects of AI deployment in SMEs. This approach would provide insights into how AI integration evolves over time and its long-term impact on business performance [14]. Understanding the phases of AI integration and the emerging challenges and benefits over time would be valuable. Comparative research across sectors and countries could highlight sector-specific challenges and best practices [15].

Additionally, exploring the role of emerging technologies such as machine learning, blockchain, and the Internet of Things in enhancing AI integration could offer a more comprehensive view of SMEs’ digital transformation [19]. Examining AI’s specific impact on various aspects of SME operations, including customer interactions, supply chain management, and product development, would deepen our understanding of how AI transforms different business functions [8]. Finally, future research should investigate the role of legislative and regulatory frameworks in facilitating or hindering AI adoption in SMEs. Understanding how government interventions can support SMEs in their digital transformation journey would be valuable [52].

Overall, this study lays the foundation for more effective AI adoption strategies tailored to the needs of SMEs. By addressing the identified challenges through strategic planning, SMEs can harness AI’s transformative potential, ensuring long-term growth and a competitive advantage in the digital age. Future research should build on these findings, offering deeper insights and practical solutions to support SMEs in their AI integration efforts.
